# ‘*Pyrococcus furiosus*, 30 years on’

**DOI:** 10.1111/1751-7915.12695

**Published:** 2017-02-20

**Authors:** Servé W. M. Kengen

**Affiliations:** ^1^ Laboratory of Microbiology Wageningen University and Research Stippeneng 4 6708WE Wageningen The Netherlands

## Abstract

*Pyrococcus furiosus* has come of age. In 1986 the first publication on a remarkable microorganism, *Pyrococcus furiosus*, appeared. Now, 30 years later it is still “the fast and the furious“.

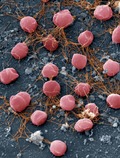

Thirty years ago, a remarkable microorganism was described for the first time by Karl Stetter and coworkers (Fiala and Stetter, 1986) (Fig. 1). Its name, meaning furious fireball, was special and over the years may have fuelled the imagination of many, but it also held a promise for the future. Now, three decades later, we can conclude that *Pyrococcus* kept that promise and that it indeed became ‘furious’ in many respects.

**Figure 1:**
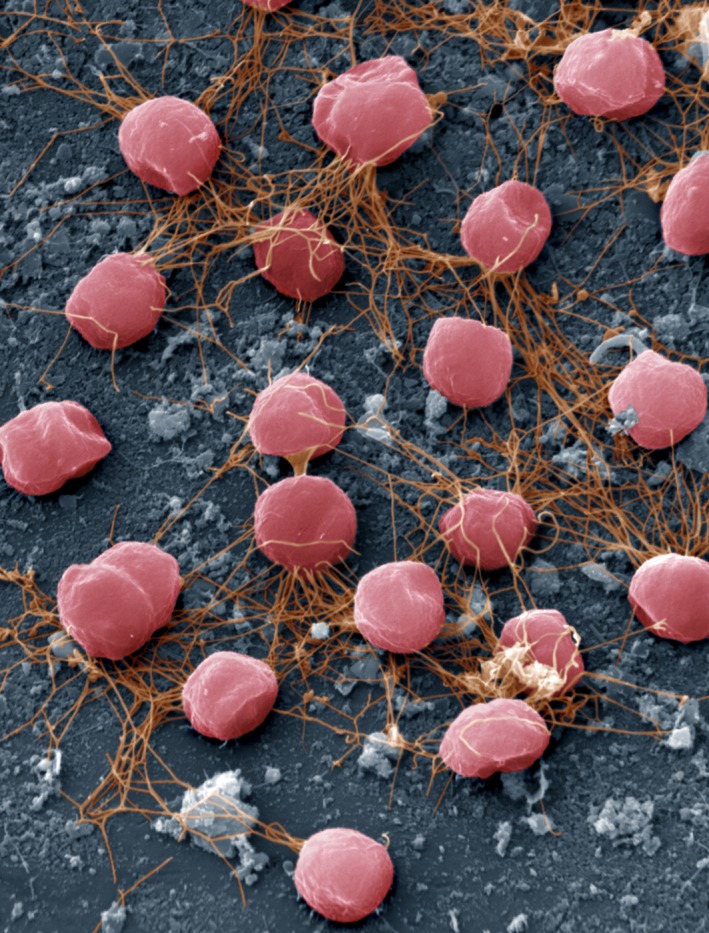
Coloured scanning electron micrograph of *Pyrococcus furiosus* cells (Photograph: ANP).

Although *Pyrococcus furiosus* was not the first hyperthermophile described to be able to thrive above the boiling point of water, it soon became one of the best studied representatives. One of the reasons was its vivid growth with a doubling time of ~37 min, which was together with its strong motility the reason for the name ‘furiosus’. Another reason was that in contrast to many other hyperthermophiles, it preferred sugars (starch) over amino acids (proteins) for its anaerobic metabolism. Whereas efficient growth on proteins required elemental sulfur as electron acceptor, producing hydrogen sulfide, growth on oligosaccharides could do without sulfur, which made culturing and harvesting of cells much easier. In particular, starch‐derived sugars were rapidly fermented to acetate, CO_2_ and H_2_ as end‐products.

Soon after its discovery in 1986, the first metabolic discoveries came to see the light of day. *P. furiosus* was one of the first archaea whose sugar metabolism was investigated in detail and it appeared that it was unlike the classical glycolytic pathways in many respects. Sugar kinases were shown to require ADP instead of ATP (Kengen *et al*., 1994) and glyceraldehyde‐3‐phosphate oxidation was not coupled to ATP synthesis and required ferredoxin instead of NAD^+^ (Mukund and Adams, 1995). Conversion of phosphoenolpyruvate to pyruvate was AMP and PPi dependent, catalysed by phosphoenolpyruvate synthase (Imanaka *et al*., 2006). Conversion of acetyl‐CoA to acetate occurred by a one‐step reaction, not involving acetyl‐P as intermediate (Glasemacher *et al*., 1997). Disposal of reductant to hydrogen involved a novel type membrane‐bound hydrogenase complex (Mbh) composed of 14 proteins (Sapra *et al*., 2000). It showed a primitive type of respiration, because it enabled the build‐up of a proton gradient (Sapra *et al*., 2003). *P. furiosus* was also shown to contain several aldehyde oxidoreductases, which appeared to contain tungsten, an element rarely used in enzymes (Roy *et al*., 2001). These are just a few early examples, but the list of novel and unusual metabolic discoveries kept on growing and is now extensive.

*Pyrococcus furiosus* not only revealed a multitude of unprecedented metabolic reactions, it also was a source of thermostable enzymes, with potential applications in various industrial processes. Probably, the most famous example is the DNA polymerase I that was already described in 1991 (Lundberg *et al*., 1991), and which possessed an associated 3′‐to‐5′ exonuclease activity. Due to this proofreading ability, this Pfu‐DNA polymerase had a much lower error rate in PCRs, compared with the Taq‐DNA polymerase. The Pfu polymerase is now used in thousands of PCRs all over the world. Various other *P. furiosus* enzymes have been isolated in the past three decades, some exhibiting extreme thermostability. For example, a β‐glucosidase had a half‐life of 85 h at 100°C (Kengen *et al*., 1993) and an α‐amylase had a half‐life of 2 h at even 120°C (Jorgensen *et al*., 1997).

Already in the early years, *P. furiosus* was called the *E. coli* of the hyperthermophiles; however, the absence of a genetic system severely hampered its potential for biotechnological applications. Chromosomal modifications were possible in the closely related *Thermococcus kodakarensis* (Sato *et al*., 2003) and two *Sulfolobus* species (Wagner *et al*., 2009), but these all had lower temperature optima. It lasted until 2010 when a plasmid‐based transformation system was developed (Waege *et al*., 2010), soon followed by chromosomal genetic manipulation of a naturally competent *P. furiosus* strain (COM1) (Lipscomb *et al*., 2011). Chromosome‐encoded expression of heterologous genes now became possible, including genes of lower temperature origin or even from bacteria. This opened up many more possibilities for metabolic engineering, including the introduction of novel pathways for alternative product formation, to respond to the requests of the pharmaceutical industry or the biobased economy. In particular, the research groups of Michael Adams (University of Georgia) and Robert Kelly (North Caroline State University) were pioneering in this respect and several of their recent achievements were remarkable and opened up new avenues for sustainable production of chemicals and fuels. Synthetic pathways have been developed for lactate (Basen *et al*., 2012), CO_2_ fixation (Keller *et al*., 2013; Hawkins *et al*., 2015), butanol (Keller *et al*., 2015), acetoin (Nguyen *et al*., 2015) and ethanol (Basen *et al*., 2014). Because most heterologous genes were derived from somewhat lower temperature ranges, a temperature shift approach was developed, in which optimal growth at ~95°C was followed by an expression phase at more moderate temperatures (Basen *et al*., 2012). Moreover, gene expression was controlled by a cold‐shock promoter, avoiding the use of chemical inducers. By this method, the Adams group demonstrated production of lactate using a bacterial lactate dehydrogenase gene (*Caldicellulosiruptor bescii*). Another, more demanding task was to confer autotropy to the typical heterotrophic *Pyrococcus*. This was accomplished by the heterologous expression of five genes of the carbon fixation cycle of the archaeon *Metallosphaera sedula*, which grows autotrophically at 73°C. The engineered *P. furiosus* strain was able to incorporate CO_2_ into 3‐hydroxypropionic acid using hydrogen gas as source of reductant (Keller *et al*., 2013; Hawkins *et al*., 2015). One of the special native features of *P. furiosus* is that it harbours a soluble hydrogenase (SHI) that is able to use hydrogen gas for the reduction of NADP, thereby providing a constant supply of reducing power for biosynthetic purposes (Keller *et al*., 2017). Another noteworthy engineering achievement is the work reported by Basen *et al*. (2014), which describes a completely novel pathway for ethanol formation (Basen *et al*., 2014). Whereas in conventional systems acetaldehyde is derived from pyruvate (yeasts) or acetyl‐CoA (fermentative anaerobes), here acetate is used as source of acetaldehyde. Acetate reduction to acetaldehyde requires low‐potential electrons (${E_{0}}^{\prime }$ ^^=^ ^−580 mV), which cannot be provided by NAD(P)H (${E_{0}}^{\prime }$ ^^=^ ^−320 mV). Therefore, the reaction requires the low‐potential electron carrier ferredoxin (${E_{0}}^{\prime }$ ^^=^ ^−500 mV) and is catalysed by an aldehyde:ferredoxin oxidoreductase (AOR), one of the native tungsten‐containing enzymes present in *P*. *furiosus*. Reduced ferredoxin is produced at two positions in the pyrococcal glycolysis. Thus, by the insertion of a single gene, *viz*. an alcohol dehydrogenase gene (AdhA) from *Thermoanaerobacter* strain X514, the groups of Adams and Kelly accomplished the conversion of glucose (maltose) to ethanol, with a yield of 90% of the theoretical ethanol yield. The AdhA is NADPH‐ dependent, which may be produced from glyceraldehyde‐3‐phosphate (GAPN) or from hydrogen using the SHI. One of the benefits of the engineered strain was that it can use alternative carboxylic acids instead of acetate, like butyrate, propionate and isobutyrate, leading to the production of the corresponding alcohols. In addition, the authors accomplished the use of carbon monoxide as alternative source of reduced ferredoxin, by introducing the genes of the CO‐dehydrogenase from *Thermococcus onnurineus*. The use of CO (via the introduced CO‐dehydrogenase) and H_2_ (via the native SHI) permits the use of syngas (a mixture of CO, CO_2_ and H_2_), which can be produced by gasification from renewable organics waste materials.

Recently, an alternative ethanol‐producing pathway was established in *P. furiosus* (Keller *et al*., 2017). Here, acetaldehyde and ethanol were produced directly from acetyl‐CoA by the introduction of a bifunctional alcohol dehydrogenase (AdhE), and acetaldehyde formation from acetate was blocked by deleting the AOR gene. It was shown that only AdhEs from two *Thermoanaerobacter* strains were functionally expressed and supported *in vivo* ethanol production. The other six AdhE homologues from different moderate thermophilic bacteria did not show significant activity. Highest ethanol levels were obtained, however, when a AdhE was combined with AdhA (61% of theoretical ethanol yield). Possibly, the activity of the AdhE, which relies solely on NADH, is insufficient and the NADPH‐dependent AdhA can compensate for this. Nevertheless, the ethanol yield of this AdhE/AdhA system was still lower than the earlier system using AOR/AdhA.

From the viewpoint of sustainable biomass fermentation, *P. furiosus* may, however, not be the ideal platform organism, because it is rather limited in its substrate range. It efficiently grows on starch polymers and starch oligomers, but it barely grows on monomeric sugars and it cannot use lignocellulosic feedstocks. Its repertoire of sugar hydrolases is rather restricted, especially when compared to the thermophilic bacteria *Thermotoga maritima* or *Caldicellulosiruptor saccharolyticus*. However, also these aspects may become targets for further metabolic engineering.

These examples show that *P. furiosos* is an excellent host for the expression of various synthetic pathways, involving genes of archaeal but also of bacterial origin and covering a broad temperature range. In addition to its native heterotrophic metabolism, it may also be used for an autotrophic metabolism, using CO_2_ and reductant from hydrogen and/or carbon monoxide. Moreover, the high growth temperature can have various advantages for industrial application, like a reduced risk of contamination, improved mixing and diffusion and lower cooling costs. Moreover, the downstream processing of various alcohols may become easier at higher temperatures. Altogether, the recent engineering successes show that hyperthermophilic *P. furiosus* is well suited for establishing various biosynthetic pathways and that the atypical ferredoxin‐based glycolysis, the unusual redox chemistry and the tungsten‐containing AORs make it stand out amongst moderate bacterial fermentatives used in chemical and fuel production. *P. furiosus* is ready for the next decade, still being ‘the fast and the furious’.

## Conflict of interest

None declared.
